# Genome editing in maize: Toward improving complex traits in a global crop

**DOI:** 10.1590/1678-4685-GMB-2022-0217

**Published:** 2023-03-03

**Authors:** José Hernandes-Lopes, Juliana Erika de Carvalho Teixeira Yassitepe, Alessandra Koltun, Laurens Pauwels, Viviane Cristina Heinzen da Silva, Ricardo Augusto Dante, Isabel Rodrigues Gerhardt, Paulo Arruda

**Affiliations:** 1 Universidade Estadual de Campinas, Genomics for Climate Change Research Center (GCCRC), Campinas, SP, Brazil.; 2 Universidade Estadual de Campinas, Centro de Biologia Molecular e Engenharia Genética, Campinas, SP, Brazil.; 3 Embrapa Agricultura Digital, Campinas, SP, Brazil.; 4 Ghent University, Department of Plant Biotechnology and Bioinformatics, Ghent, Belgium.; 5 VIB, Center for Plant Systems Biology, Ghent, Belgium.; 6 Universidade Estadual de Campinas, Instituto de Biologia, Departamento de Genética, Evolução, Microbiologia e Imunologia e Evolução, Campinas, SP, Brazil.

**Keywords:** CRISPR/Cas, maize transformation, transformation recalcitrance, multiplex genome editing, promoter editing

## Abstract

Recent advances in genome editing have enormously enhanced the effort to develop biotechnology crops for more sustainable food production. CRISPR/Cas, the most versatile genome-editing tool, has shown the potential to create genome modifications that range from gene knockout and gene expression pattern modulations to allele-specific changes in order to design superior genotypes harboring multiple improved agronomic traits. However, a frequent bottleneck is the delivery of CRISPR/Cas to crops that are less amenable to transformation and regeneration. Several technologies have recently been proposed to overcome transformation recalcitrance, including HI-Edit/IMGE and ectopic/transient expression of genes encoding morphogenic regulators. These technologies allow the eroding of the barriers that make crops inaccessible for genome editing. In this review, we discuss the advances in genome editing in crops with a particular focus on the use of technologies to improve complex traits such as water use efficiency, drought stress, and yield in maize.

## Introduction

As the global population grows, there is an increasing urgency for large-scale sustainable food production. The world population will reach 10 billion people by 2050, and although food production needs to increase proportionally, the ever-increasing effects of climate change threaten modern agriculture in an unprecedented manner (Gornall et al., 2010; Brás et al., 2021). Maize (*Zea mays* L.) is one of the most important crops in the world, extensively used as food, feed, fuel, and raw material by several industries (Andorf et al., 2019). Importantly, variations in temperature, precipitation, and their interaction historically have had a large impact on global yields of maize and most other major crops (Lobell et al., 2011; Ray et al., 2015; Daryanto et al., 2016). As significant and recent examples, Brazil (the world’s third largest maize producer) experienced a reduction in its maize production of approximately 18 Mt and 23 Mt in the 2015/16 and 2020/21 growing seasons, corresponding to losses of approximately 21% compared to the 2014/15 and 2019/20 seasons, respectively (CONAB, 2021). Considering the average maize price from 2018 to 2021 (CEPEA-ESALQ, 2021) these unrealized yields correspond to economic losses of approximately USD 3 and USD 5 billion. These crop failures occurred in years marked by pronounced drought (INMET, 2021), and resulted in poor yields in many of the largest producer geographies. Likewise, the 2012 drought in the U.S. (the world’s largest producer) resulted in similar yield reductions and spiking prices (Boyer et al., 2013). Thus, the continuous development of new maize cultivars aiming at better genetic adaptation, along with the adoption of improved agricultural practices, is crucial to minimize future losses resulting from the increased frequency, severity, and duration of stresses associated with global climate change (IPCC, 2022).

Yield and abiotic stress tolerance are complex traits usually strongly affected by the environment and associated with small-effect genomic *loci*. This complexity challenges dissecting the molecular mechanisms of gene actions and accurately measuring phenotypes, making it difficult to use genomic engineering tools to develop superior cultivars for those traits. Transgenic maize cultivars aiming at increased insect and herbicide tolerance have been on the market for decades, in contrast to only a few examples developed for complex traits (Yassitepe *et al*., 2021). The difficulty of applying a transgenic approach to manipulate complex traits stable in several environments has limited the development of biotech cultivars that could be widely used (Simmons et al., 2021). However, recent advances in genome editing based on CRISPR/Cas (Clustered Regularly Interspaced Short Palindromic Repeats/CRISPR/associated proteins) technologies allow simultaneous mutations on multiple genes. Additionally, coupling genome editing with haploid induction increases the possibilities for genetically engineering complex traits across germplasms used in plant breeding programs.

Most genome editing studies in maize have been performed using genetic transformation protocols based on *Agrobacterium tumefaciens* or biolistic delivery methods in a couple of temperate genotypes suitable for *Agrobacterium* infection and regeneration (Kausch et al., 2021a; Yassitepe *et al*., 2021). However, the lack of a transformation protocol applicable to a wider range of genotypes and the typical low transformation efficiency hamper the broad application of genome editing in maize. Recently, solutions to overcome these constraints have been proposed, such as using morphogenic regulators (MRs) and *in trans* genome editing. Such approaches could be broadly used in maize breeding programs worldwide, including programs based on tropical germplasm. In this review, we discuss current advances in multiplex genome editing, base and prime editing, morphogenic regulators, and *in trans* genome editing tools that could potentially be applied to engineer complex traits.

Powerful genome editing toolkits for plant breeding

Although plant breeders have relied on molecular biology tools for introducing relevant agronomic traits into elite germplasm, transgenic approaches have limitations, such as the integration of foreign DNA into random sites of the host genome, which may raise regulatory concerns. Furthermore, developing new commercial genetically modified (GM) cultivars often requires a long and costly deregulation process, limiting this endeavor mostly to large multinational companies (Schmidt et al., 2020; Whelan et al., 2020). Additionally, despite their significant beneficial impact on modern agriculture, the public still strongly rejects transgenic crops (Schmidt *et al*., 2020; Woźniak et al., 2021).

In this complex scenario, genome editing (GE) via CRISPR/Cas systems stands out as the most promising tool for the rapid development of new improved crop cultivars (Chen et al., 2019). This system’s accuracy in targeting specific sites, the opportunity to concomitantly alter multiple genes, and the possibility of segregating the CRISPR machinery by crossing while maintaining the edited *loci* are transforming plant breeding. In addition, CRISPR-based GE presents advantages over classical breeding and transgenic approaches, such as the opportunity to avoid GMO regulation by creating alleles indistinguishable from those produced by natural means, the possibility of rapidly developing stable homozygous lines mutated at precise *loci*, and relatively easy stacking of multiple advantageous traits via multiplex strategies.

Even though the original CRISPR/Cas system became a solid toolkit that revolutionized plant research and breeding, limitations such as the stochastic nature of the induced mutations prevented its application to specific cases. However, novel CRISPR/Cas-based technologies are constantly being developed, including the use of other Cas nucleases, such as Cas12a (also known as Cpf1), modified Cas nucleases with increased efficiency, deactivated Cas (dCas) or Cas nickases (nCas), chemically modified sgRNAs, and fusion of nCas or dCas to functional domains of other proteins (Chen et al., 2019; Anzalone *et al*., 2020; Gao, 2021). In addition, some CRISPR-based technologies such as base and prime editing allow for specific and precise modifications at the target *locus*, minimizing the randomness of indels caused by the non-homologous end joining (NHEJ) pathway (Anzalone *et al*., 2020; Molla et al., 2021).

Additionally, improving CRISPR/Cas-based methods involves creating new modes of genome editing and broadening their applicability to plant breeding, allowing the improvement of traits controlled by multiple genes or even developing multiple traits at once. Next, we discuss some of these techniques and their potential application for plant breeding, summarized in Table 1.

**Table 1 -:** Advantages and limitations of diverse genome editing strategies and examples of their application in maize.

Approach	Advantages	Limitations	Examples in maize
Multiplex genome editing	- Rapid stacking of multiple traits - Improvement of complex traits in few generations - Efficient strategy for gene discovery	- Larger vectors may lower transformation efficiency - Different sgRNA efficiencies may result in unequal mutation rates - Likely increase in off-target mutation rates	Zhang et al., 2020; Liu et al., 2021; Gong et al., 2021; Liu *et al*., 2022; Lorenzo et al., 2022
Base editing	- Precise and specific point mutations - Good option for editing regulatory sequences	- Less applicable to generate gene knockouts	Li et al., 2020
Prime editing	- Precise point and small mutations - Good option for editing regulatory sequences	- Still very low efficiency in plants - Guide design (pegRNA) more complex than standard sgRNA - Optimization of many variables are still required	Jiang et al., 2020
Promoter editing	- Allows strategies for increasing or altering expression patterns - Avoids modifications in protein-coding sequences	- Complexity of regulatory sequences may result in poor predictability of outcomes	Shi et al., 2017; Liu et al., 2021
*In-trans* genome editing	- Circumvents transformation recalcitrance - May generate monoallelic mutations when combined with haploid inducer strategy (HI-Edit / IMGE)	- Lower editing efficiency when compared to approaches based on stable transformation	Li et al., 2017; Kelliher et al., 2019; Wang B et al., 2019; Qi et al., 2020

Multiplex genome editing

Small contributions of several genes determine important agronomic traits such as yield. Improvement of these complex quantitative traits requires hard and time-consuming selection and multi-crossing programs, which can take years before resulting in the development of new elite cultivars, even using modern techniques such as Genome Prediction, for example. CRISPR/Cas-based multiplex genome editing (MGE) enables the creation of new lines carrying multiple genome modifications in a few generations. This possibility represents an enormous advance over traditional plant breeding and transgenics, facilitating the stacking of advantageous traits (Najera et al., 2019).

**Figure 1 -:**
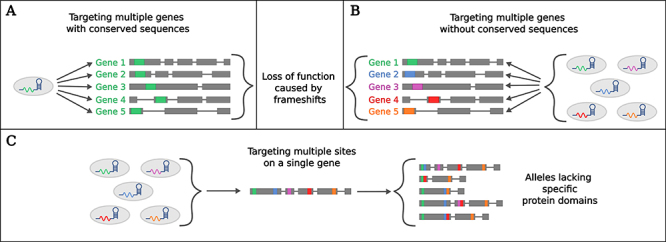
Multiplex approaches for genome editing of multiple genes or multiple sites of a single gene. **A**. A single sgRNA is designed to target multiple genes with conserved domains/sequences (green boxes). **B**. Multiple sgRNAs are designed and simultaneously delivered, targeting sequences in different genes (colored boxes). **C**. Multiple sgRNAs may target different sections of a single gene, resulting in the deletion of specific regions between the target sites.

MGE can be used to target similar and/or dissimilar sequences. For example, a single sgRNA can be used to target multiple genes with conserved regions (Figure 1A), whereas more than one sgRNA can target multiple genes (Figure 1B) or even multiple sites on the same gene (Figure 1C) (Najera et al., 2019).

MGE is one of the most promising GE methods to improve complex traits. For example, by simultaneously knocking out the genes *GW2*, *GW5*, and *TGW6*, which are responsible for decreasing grain weight, new rice lines were created with ~30% higher grain weight (Xu et al., 2016). Additionally, by segregating the CRISPR machinery through crossing, transgene-free GE progeny was obtained, highlighting the advantage of this technique in developing new plant cultivars (Xu *et al*., 2016). In another example, wheat grain length and weight were increased by targeting a conserved region of three homologs of the *TaGASR7* gene with a single sgRNA. Transgene-free plants knocked out for all six alleles were obtained in a single generation using a transient expression setting for the CRISPR machinery (Zhang et al., 2016).

Different MGE strategies have been used in maize. For instance, Qi et al. (2016) proposed an optimized tRNA-processing system-based method in maize in which up to four sgRNAs can be inserted into an array. The endogenous tRNA-processing system not only successfully processes the primary transcript from a more compact expression cassette but also seems to boost editing efficiency (85.7%-100%). The increase in editing efficiencies may be due to the A- and B-boxes in the tRNA sequences, which recruit transcription factors (White 2011; Xie et al., 2015; Minkenberg et al., 2017). This result is interesting for maize GE since high editing efficiency is crucial when crop transformation efficiency is low, mitigating this considerable bottleneck. MGE in maize can also be performed with a larger number of sgRNAs. For instance, vectors harboring up to twelve individual sgRNA expression cassettes have been successfully used for the transformation of an “editor” (i.e., Cas9-expressing) maize line (Lorenzo et al., 2022). Gong et al. (2021) compared the CRISPR/Cas12a versus the CRISPR/Cas9 system for MGE targeting the maize bZIP transcription factor *Opaque2* (*O2*). Although CRISPR/Cas12a showed lower editing efficiency than CRISPR/Cas9 in the T_0_ and T_1_ generations, it led to a greater mutation variety in T_2_. In addition, the editing efficiency of the Cas12a-based system was positively correlated with the nuclease expression level, proving to be a valuable alternative for MGE in maize.

Although not yet broadly applied to maize, there are some reports of MGE applied to investigating and/or improving complex traits in this crop. For example, plant stature significantly impacts crop production, so the development of short-stature cereals was the foundation for the Green Revolution (GR) (Peng et al., 1999). The dwarf and semidwarf cultivars have been shown to present several benefits, such as increased resistance to lodging caused by wind and rain, easy management in the field since plants are shorter and thus more accessible, and increased yield given that plants are more compact and require a smaller cultivated area. It has been shown that the application of gibberellin biosynthesis inhibitors during maize development leads to reduced plant height and improves water use efficiency and harvest index (grain mass to aboveground total mass ratio) (Hütsch and Schubert 2018). However, developing new plant lines with short stature by traditional breeding can be an overly long process. For example, 10 generations of directional selection were necessary to achieve maize plants with desirable heights (Teixeira et al., 2015). In a simpler approach, semidwarf maize was generated by the knockout of *ZmGA20ox3* using two sgRNAs (Zhang et al., 2020).

MGE was also used for targeting multiple genes in maize. Because most cultivated maize plants are hybrids, detasseling is important for preventing self-pollination. Thus, identifying potential genes leading to male sterility is of great interest. Although several homologs of such genes are already known in maize, they often belong to families comprising genes with redundant functions. Through an MGE approach, Liu et al. (2022) were able to identify genes that can lead to male sterility when knocked out either individually or in combination with others. This study opens the possibility of selecting specific combinations of gene knockouts for establishing commercial lines.

Another interesting approach for MGE in maize has been recently described. The technique, dubbed BREEDIT, aims at improving complex traits controlled by many genes, such as drought resistance and yield. In BREEDIT, populations transformed with different sets of sgRNAs (twelve different sgRNAs on each set) are then crossed, stacking up mutations in an increasing number of genes. This approach is useful for both trait improvement as well as for the discovery of new genes contributing to a given trait of interest (Lorenzo et al., 2022).

Taken together, these works demonstrate the potential of MGE for the rapid improvement of complex traits, especially those controlled by numerous genes. It is important, however, to keep in mind the limitations of this approach. For example, designing a set of sgRNAs for specific genes belonging to conserved families can be challenging. Genotyping many genes can also require more sophisticated sequencing approaches, such as amplicon sequencing. Adding many sgRNAs into one vector can also potentially reduce transformation efficiency. An alternative method that circumvents the obstacles of managing excessively large plasmids is the direct delivery of ribonucleoprotein (RNP) complexes. Such RNPs are synthesized *in vitro* by combining Cas9 with mature sgRNA molecules (Woo et al., 2015; Svitashev et al., 2016; Liang et al., 2017). This approach is transgene-free since no foreign DNA is delivered to the plant. Successful RNP-mediated genome editing has also been shown for maize immature embryos co-bombarded with sgRNA:Cas RNPs, along with transformation-enhancing genes (morphogenic regulators) and a selectable/visible marker (MoPAT-DsRED) (Svitashev *et al*., 2016; Liang *et al*., 2017). This promising result indicates the potential of using RNPs for MGE in maize while paving the way for its application in crops not prone to protoplast regeneration. Even though other CRISPR/Cas methods may generate transgene-free cultivars, crosses are needed to eliminate the exogenous editing system. In the case of RNPs, edited events will most likely bypass regulatory hurdles without additional efforts.

Promoter editing

**Figure 2 -:**
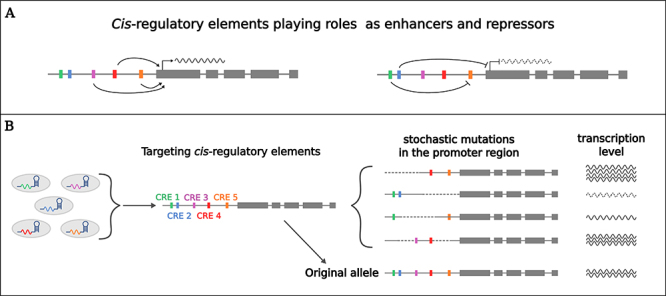
Using CRISPR/Cas to edit the promoter region of target genes. **A**. *Cis* -regulatory elements (CREs) present upstream of a given gene may act as enhancers (purple, red, and orange boxes) or repressors (green and blue boxes), modulating gene expression. **B**. Multiplex genome editing approach: multiple sgRNAs targeting different CREs may result in stochastic mutations in the promoter region, resulting in alleles with different expression patterns/levels. This method may ultimately lead to lines with a phenotypic gradient.

Although coding sequences are usually the targets of choice for GE, other genetic elements, such as regulatory sequences, can also be targeted to modulate spatiotemporal gene expression patterns. For instance, genes playing essential roles in plant domestication have more stable expression patterns in cultivated species than in their wild relatives, suggesting that specific *cis-*regulatory elements (CREs) were selected during domestication (Lemmon et al., 2014; Swinnen et al., 2016). Thus, regulatory sequences can be targeted by GE to fine-tune gene expression levels and tissue preference to create an array of subtle phenotypes (Figure 2) (Wittkopp and Kalay, 2012; Swinnen *et al*., 2016; Ganguly et al., 2022).

Other non-coding regions can also be targeted for regulating expression, such as introns and upstream open reading frames. Nevertheless, non-coding region editing is far from trivial. Given the complexity of the CRE landscape and mode of action, phenotypic effects resulting from mutations within promoter regions are hardly predictable (Rodríguez-Leal et al., 2017). Compensation effects such as those from enhancers and repressors and the distance between different CREs contribute to this lack of predictability. Another layer of complexity comes from the chromatin conformation in the promoter region, with epigenetic modifications and chromatin accessibility also controlling gene expression levels (Rodgers-Melnick et al., 2016; Schmitz et al., 2022).

Even though editing promoters being challenging, it holds great potential for plant breeding by facilitating the approval of new commercial varieties. Promoter editing allows manipulating a small number of nucleotides in non-coding regions without exogenous DNA in the final product; therefore, it may overcome the lengthy and costly regulatory processes and social rejection hurdles that come with transgenic events (Lassoued et al., 2019). Furthermore, promoter GE may allow manipulating quantitative traits, adding diversity to plant breeding. As an example, tomato lines with fruits presenting a gradient in the number of locules were developed through MGE targeting the promoter region of the *SlCLV3* gene (Rodríguez-Leal et al., 2017). Similarly, an allelic series of the maize *ZmCLE7* gene was generated by promoter GE, resulting in lines presenting variability for inflorescence meristem size, ultimately leading to enhanced grain-yield-related traits (Liu et al., 2021). Grain yield per ear was also recently improved by both knocking out and promoter editing of the *ZmACO2* gene. In this case, the reduced expression / loss-of-function of *ZmACO2* resulted in increased yield in different genetic backgrounds, including hybrids (Ning et al., 2021). 

In a noteworthy example addressing a highly complex trait, maize plants with increased drought tolerance were generated by either introducing or swapping the promoter region of the *ARGOS8* gene with the endogenous and stronger promoter from *GOS2*, which contains drought-responsive *cis*-elements (Shi et al., 2017). *ARGOS8* negatively regulates ethylene responses (Shi *et al*., 2016); therefore, its overexpression promotes cell expansion and/or division, enhancing plant growth and mitigating yield loss under drought (Shi *et al*., 2017). Importantly, similar increases in grain yield had already been reported in transgenic maize plants overexpressing *ARGOS8* when submitted to drought conditions (Shi *et al*., 2015), underscoring the potential of promoter-editing strategies to mimic desirable phenotypic effects originated from a transgene-mediated expression of target genes.

The environment has a great impact on the expression patterns of genes underlying complex traits, such as enhanced yield and tolerance to various stresses, but the same genes and pathways they act upon are also often required for plant growth and development. Because classical biotechnological approaches for the manipulation of such genes rely on overexpression or loss-of-function knockouts, they frequently result in pleiotropic effects and undesirable tradeoffs (Huot et al., 2014). Thus, fine-tuning their expression patterns via GE of regulatory sequences holds great potential for improving complex traits.

Base and prime editing

Other CRISPR/Cas strategies at the forefront of plant science that have great potential for breeding applications regard base and prime editors. Base editing relies on a fusion between a catalytically impaired Cas9 (nCas9 or dCas9) with a cytosine or adenosine deaminase. The modified Cas9 guides and anchors the fused protein to the target sequence driven by the sgRNA. Then, the fused protein can change the DNA sequence in a programmable manner without creating double-strand breaks (DSBs) (Komor et al., 2016; Gaudelli et al., 2017; Anzalone *et al*., 2019).

Among the first tools for base editing are the adenine base editors (ABEs), which allow A-to-G (or T-to-C) transitions (Gaudelli et al., 2017), and cytidine base editors (CBEs), which promote C-to-T (or G-to-A) conversions (Komor et al., 2016). New recently developed base editors allow C-to-G (CGBEs) and C-to-A conversions in mammalian and bacterial cells, respectively (Molla et al., 2021). While C-to-A conversions are still restricted to bacterial cells, CGBEs have been tested in rice, tomato, and poplar (Sretenovic et al., 2021), but efficiencies are still very low.

Base editing has already been used in many crops, such as rice, maize, wheat, potato, tomato, watermelon, and cotton (Zong et al., 2017; Li et al., 2018; Mishra et al., 2020; Qin et al., 2020). For maize, targeted conversion of C-to-T in *ZmALS1* and *ZmALS2* genes, with an efficiency of up to 14%, generated transgene-free edited plants harboring a homozygous mutation for *ALS1 or* double mutation for the two *ALS* genes, leading to herbicide-tolerant plants (Li *et al*., 2020).

Further possible programmed and precise sequence alterations are possible with prime editing, which allows insertions (up to 44 bp), deletions (up to 90 bp), and single base alterations, including all 12 possible base-to-base conversions, without requiring DSBs or donor DNA templates (Anzalone *et al*., 2019; Zong et al., 2022). The prime editing system is based on a Cas nickase fused to an engineered reverse transcriptase and programmed with a prime editing guide RNA (pegRNA). The pegRNA both specifies the intended cut site (primes with the target DNA) and acts as a template (encodes the desired edit) for precise editing at the target genomic locus (Anzalone *et al*., 2019; 2020). A reverse transcriptase extends the target DNA sequence based on the pegRNA and this new strand competes with the original one to bind with the non-target DNA strand. If the edited strand anneals, a mismatch occurs. Since the non-target strand is nicked by nCas9, it is more likely that the cell will copy the newly edited strand to repair the damaged DNA (Marzec et al., 2020).

Prime editing, first proposed by Anzalone *et al*. (2019) to edit the human genome, has already been successfully employed to edit plant genomes, although with very low efficiencies (Tang et al., 2020; Xu R et al., 2020; Xu R *et al*., 2020; Lin et al., 2020; 2021). Improvements such as modifications of the reverse transcriptase functional domains, which lead to an average of 5.8-fold increase in editing efficiency compared to the original prime editor, have been recently reported (Zong et al., 2022). An independent improvement was achieved in rice, maize, and human cells by fusing the reverse transcriptase to the Cas9 N-terminal instead of the C-terminal and multiple-nucleotide substitutions in the reverse transcriptase template to enhance prime editing efficiency (Xu *et al*., 2022). 

Base and prime editing are of special convenience for developing traits known to arise from point mutations. For example, prime editing was applied to generate sulfonylurea herbicide-resistant maize (Li et al., 2020), tomato, potato (Veillet et al., 2019), and oilseed rape (Wu et al., 2020). Jiang et al. (2020) also generated double mutations in*ZmALS1*and*ZmALS2* using prime editors in maize, with higher efficiency than previously reported. Moreover, base and prime editing can also be helpful to disrupt or even introduce regulatory sequences to alter gene expression (Molla et al., 2021). For example, in strawberry (*Fragaria vesca*), base editing of an upstream open reading frame (uORF) led to the development of lines showing a continuum in sugar content (Xing et al., 2020).

Although with less obvious applications to complex traits, these examples of base and prime editing highlight the importance of such technologies to develop new maize cultivars. While prime editing opens the possibility of inducing precise mutations in coding or regulatory sequences, major improvements in editing efficiency are still required before the technique can be largely applied to maize.

### Limitations and strategies for the application of genome editing in maize breeding

Despite its great potential for plant breeding, CRISPR-based GE faces important obstacles that hinder its large-scale application to deploy new commercial maize lines. Given the high specificity of the CRISPR/Cas systems, sequencing of the target sites is required for each different genotype to be edited, ensuring the correct design of sgRNAs. Recently, this obstacle has been somehow alleviated with the publication of the genome of 26 maize inbred lines used as founders for the maize nested association mapping population (Hufford et al., 2021). This resource may not only help sgRNA design but also hint at *loci* associated with relevant agronomic traits, representing potential targets for genome editing. However, it is still advisable to re-sequence target *loci* from the explant-donor lines, as even these genotypes can present some polymorphisms that could interfere with editing efficiency. Sequencing the target genotypes would also help in identifying potential off-targets.

One of the main bottlenecks for applying biotechnological tools in maize breeding is the recalcitrance of maize lines to *Agrobacterium*-mediated transformation. Although there are some maize lines amenable to this standard transformation method, such as Hi-II and B104, most of these lines are not suitable for proper field trials of GE events and/or for commercial application (Kausch et al., 2021a; b; Yassitepe *et al*., 2021). In addition, even among these transformable maize lines, transformation efficiency with standard protocols is usually low. Taken together, the low efficiency of some GE techniques in plants (e.g., homology-directed repair and prime editing) and the recalcitrance of most maize genotypes to transformation hamper GE application for maize breeding. For instance, one of the most studied maize lines, B73, which is also the reference maize genome, is highly recalcitrant to standard transformation protocols (Andorf et al., 2019; Yassitepe *et al*., 2021).

Cultivated maize plants are usually hybrids between parental elite inbred lines. These inbred lines are genetically homozygous and harbor alleles encoding important agronomic traits. Hybrids obtained by crossing distinct inbred lines perform better in the field due to a phenomenon known as heterosis (Li et al., 2021). Thus, breeding programs routinely cross different inbred parents to produce new F_1_ hybrid combinations. Although very successful for classical plant breeding, cultivated hybrids represent an additional obstacle to applying GE in maize. For example, for a trait resulting from a gene knockout, both parental lines need to be edited, which is usually unfeasible owing to genotype recalcitrance to transformation. Furthermore, even though hybrids are widely used in crop breeding to capture heterosis, their phenotypic superiority obtained in F_1_ is lost in further crosses. 

Maize transformation has been extensively reviewed from different perspectives (Kausch et al., 2021a,b; Yassitepe *et al*., 2021). Several strategies to overcome the low transformation efficiency, genotype dependency, and time-consuming introgression of GE alleles have been reported. Two promising systems involve improved transformation protocols based on the ectopic expression of morphogenic genes, which help in the regeneration process, and methods for *in trans* genome editing in a transformation-free manner. In addition, breakthroughs in synthetic apomixis are promising for fixing hybrid vigor in GE lines.

Morphogenic regulators: groundbreaking tools toward “universal” transformation protocols

A promising strategy to overcome the recalcitrance of most maize genotypes to genetic transformation is the introduction of morphogenic regulators (MRs) in the construct designed for genetic transformation. The most frequent MRs used in maize are the transcription factors *BABY BOOM* (*BBM*) and *WUSCHEL* (*WUS*) (Lowe et al., 2016; Mookkan et al., 2017; Lowe *et al*., 2018; Barone et al., 2020; Aesaert et al., 2022). When co-expressed, *BBM* and *WUS* can induce somatic embryogenesis in several plant tissues, including the scutellum of immature zygotic embryos (currently the most efficient starting material for maize transformation). This method reduces the time required for tissue culture since it skips the callus-forming stage by directly inducing somatic embryogenesis (Lowe *et al*., 2016; Lowe *et al*., 2018). However, the most significant advantage of such a technique relies on its ability to induce somatic embryogenesis in genotypes otherwise recalcitrant to tissue regeneration (Lowe *et al*., 2016, 2018; Masters et al., 2020).

Because constitutive expression of MRs impairs normal plant development, MR expression must be confined to the embryogenesis induction phase. For this, MR expression cassettes are either excised from the T-DNA after somatic embryogenesis or only transiently expressed soon after transformation. Auto-excision is usually achieved by a recombination system such as the CRE/loxP (Lowe et al., 2016; Mookkan et al., 2017; Zhang et al., 2019; Masters et al., 2020; Aesaert et al., 2022), while other strategies rely on the delivery of MRs in a separate plasmid designed not to be integrated into the genome (Svitashev et al., 2016; Hoerster et al., 2020). Choosing appropriate promoters for MRs also improved the quality of recovered transformed events. It can even eliminate the need to remove the MR cassette from the T-DNA (Lowe *et al*., 2018).

In their seminal work, Lowe et al. (2016) recovered transgenic quality events from four recalcitrant maize lines at a frequency of up to 13.7%. Similar results were obtained for the reference genotype B73, with a transformation frequency of approximately 15% (Mookkan et al., 2017). Moreover, at least 22 DuPont Pioneer’s inbred lines were responsive to the MR-based transformation protocol, indicating that it may indeed be genotype-independent (Lowe *et al*., 2018). More recently, the technique was applied to B104, a line commonly used for transformation in academic settings. By fine-tuning the media culture and including an MR expression cassette in the T-DNA, the transformation efficiency of B104 increased from 1% to 5% (Aesaert et al., 2022).

Other recent strategies use a different set of MRs. *GROWTH REGULATING FACTOR 5* (*GRF5*) and a *GRF4/GRF-INTERACTING FACTOR 1* (*GIF1*) fusion has been reported to improve regeneration efficiency in monocot and dicot plants (Debernardi et al., 2020; Kong et al., 2020). These morphogenic genes are involved in organ development, which may represent an advantage over *BBM*/*WUS* constructs since their continued expression does not culminate in developmental penalties for the transformed plant. Overexpression of putative *GRF5* maize orthologs increased the transformation efficiency of the A188 inbred line by approximately 3-fold (Kong *et al*., 2020), while the GRF4-GIF1 fusion not only increased the number of regenerated plants in rice, triticale, and wheat but also increased the number of wheat genotypes amenable to transformation (Debernardi *et al*., 2020).

“Transformation-free” methods for genome editing

In the case of *in trans* genome editing, methods including the desired-target mutator (DTM) (Li et al., 2017), haploid induction (HI) editing technology (HI-Edit) (Kelliher et al., 2019), and haploid-inducer mediated genome editing system (IMGE) (Wang B et al., 2019) may accelerate the development of edited maize varieties. These technologies allow delivering the CRISPR/Cas machinery directly to transformation-recalcitrant lines by crossing elite inbred lines with a stably transformed line harboring a CRISPR/Cas construct, allowing the continued activity of CRISPR/Cas to generate new mutated alleles. Thus, although not completely avoiding genetic transformation, DTM and HI-Edit/IMGE limits the transformation step to amenable genotypes (recently reviewed by Gao 2021; Gu et al., 2021; Yassitepe *et al*., 2021; Impens et al., 2022).

DTM was first used to generate newly edited alleles of the *LIGULELESS1* (*LG1*) gene in maize (Li et al., 2017). Crossing T_1_ plants harboring CRISPR machinery with six different maize lines resulted in over 20% mutated alleles in the progeny. As a breeding scheme, this method requires a series of backcrosses with marker-assisted selection to integrate the new alleles into an elite line for hybrid production. However, this approach avoids the linkage drag effect and uses a smaller population size to recover the recurrent genome, increasing the precision and time to introduce a new allele into elite lines (Li *et al*., 2017). DTM was also applied to induce knockout mutations of*ZmWX*, resulting in hybrids with high endosperm amylopectin content (Qi et al., 2020). A series of mutated alleles were generated in both hybrid parent lines. After two backcrosses, lines from both parents showed more than 87% of the genome recovered and ~ 20% more amylopectin content than their wild-type counterparts (Qi *et al*., 2020).

**Figure 3 -:**
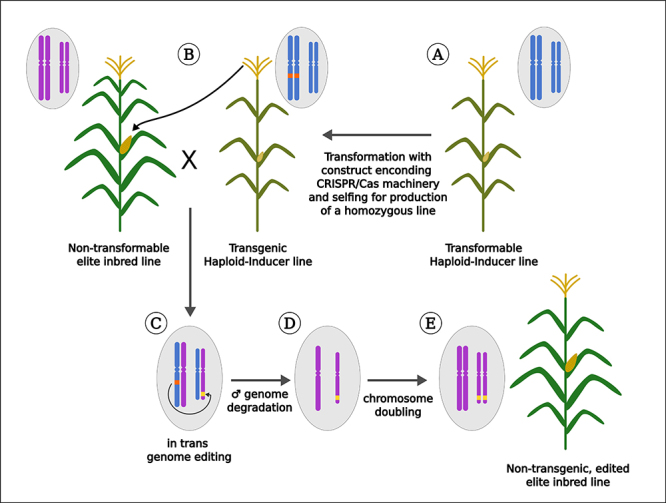
*In trans* genome editing in maize. First, a haploid-inducer (HI) line (amenable to transformation) is equipped with the CRISPR/Cas machinery targeting a specific *locus* (**A)**. Next, HI pollen is used to pollinate plants from a non-transformable genotype (**B)**. After fertilization, the CRISPR/Cas machinery encoded by the male parental genome edits the female genome (**C)**. The male genome is degraded, resulting in a haploid embryo containing only the female genome (**D)**. Chromosome doubling is achieved by applying chemical agents, resulting in a non-transgenic double haploid plant harboring the edited female genome (**E)**.

Although the DTM strategy was an advance on genome editing approaches for maize breeding, especially by avoiding the linkage drag effect, it still requires a series of backcrosses to integrate the newly edited allele on an elite line. An advance in the *in trans* genome editing strategy was reported a couple of years later by two research groups, integrating genome editing with haploid induction (Hi-Edit) (Kelliher et al., 2019) and the haploid-inducer mediated genome editing system (IMGE) (Wang B et al., 2019). The use of double haploid (DH) in maize breeding is a common practice in several private and public breeding programs by eliminating generations of self-pollination for inbred production (Chaikam et al., 2019). In HI-Edit/IMGE methods, the CRISPR/Cas machinery is introduced into a haploid inducer line that is used to pollinate maternal elite lines. In the haploid progeny, the paternal genome carrying the CRISPR/Cas9 transgene is eliminated. However, in a fraction of these haploids, the maternal genome is edited *in trans*. The next step is to treat the haploid progeny with a chromosome-doubling agent to produce DH lines. Then, these lines are screened for the presence of new edits, which are immediately monoallelic (homozygous) (Figure 3). For instance, genome-edited knockouts for the *ZmLG1* and *UB2* genes were generated and crossed with a haploid inductor line, resulting in edited haploid progenies at 3-4% efficiency (Wang B *et al*., 2019). In another example, a haploid inductor edited line for the *MTL* pollen-specific phospholipase gene was generated and used to pollinate different maize lines, successfully generating GE double haploids (Kelliher *et al*., 2019).

In addition to being a transformation-free method, the Hi-Edit/IMGE approach generates transgene-free edited plants, an important aspect for GM-unfriendly markets. Further developments in the Hi-Edit/IMGE methods have been published, mainly to improve the haploid induction rate (Kelliher et al., 2017, 2019; Zhong et al., 2019), identify seeds with haploid embryos by modifying visual traits such as anthocyanin biosynthesis (Chaikam et al., 2019) or integrate visible transgenic markers into the inducer lines (Yu and Birchler 2016; Yan et al., 2021; Xu et al., 2021). Thus, *in trans* genome editing methods can precisely introduce desired traits into the genome of elite lines, overcoming the time-consuming traditional introgression efforts. As a result, they can also increase allele variability, which is potentially beneficial for future breeding demands.

Synthetic apomixis and its potential role for maize GE

Clonally propagating maize hybrid F_1_ seeds could be used to maintain the heterotic progeny resulting from inbred crosses, facilitating hybrid seed production. This purpose can be achieved by engineering apomixis, an asexual reproductive pathway that gives rise to offspring identical to the maternal plant (Wang C et al., 2019). Apomixis-based systems can be developed to induce the formation of unreduced gametes in the ovule and a two-step approach based on converting meiosis into mitosis, followed by eliminating the paternal genome has been tested in some plant species. For instance, a Mitosis instead of Meiosis (MiMe) technology was developed in rice by stacking mutations in the meiotic genes *REC8*, *PAIR1*, and *OSD1* (Mieulet et al., 2016). More recently, F_1_ heterozygosity was fixed in rice by recreating the MiMe genotype via CRISPR/Cas9. Such genotype produces diploid gametes and tetraploid seeds (Wang C *et al*., 2019). Further mutation of the haploid induction *MATRILINEAL* (*MTL*) gene led to paternal genome elimination. The MiMe rice plants without the paternal genome produce self-fertilized F_1_ hybrids and clonal seeds with the same ploidy and heterozygous genotype. A similar result was obtained by combining the MiMe triple mutant with ectopic expression of the *BBM1* transcription factor in the egg cell, which can trigger parthenogenesis. This approach resulted in clonal progeny that retained the asexual-propagation capability for multiple generations (Khanday et al., 2019).

Despite the recent advances in synthetic apomixis in monocots such as rice, the process is still poorly explored in maize, in which efforts are more focused in the development of haploid induction. Apomixis-based genome editing strategies can be exploited to directly edit hybrids, fix hybrid vigor and facilitate clonal propagation of GE lines, although they need to be further optimized to be vastly applied in hybrid seed production in various crops (recently reviewed by Ozias-Akins and Conner 2020; Chen et al., 2021; Yin et al., 2022).

## Concluding Remarks

The development of new maize cultivars incorporating complex quantitative traits modified by biotechnological approaches can be a reality in the near future, considering the recent advances in genome editing. Although biotech maize resistant to insects and herbicides has dramatically impacted maize production worldwide, the improvement of complex quantitative traits such as drought and heat tolerance, nutrient acquisition and use efficiency, and yield are imperative to meet the ever-growing demand for maize-derived products. Currently, maize breeding programs have a wide range of unprecedented available tools to help speed up and accurately develop new cultivars. These tools include high-throughput phenotyping, advanced statistics and computational methods, crop models, new genome sequencing and predictions, and rising genome editing approaches. Here, we presented and discussed some basic concepts and emerging genome editing tools for maize and other crops. There are still critical challenges and questions to be addressed before the massive application of genome editing tools to maize breeding is attained. However, because genome-edited cultivars can be already accepted as non-GM in countries that produce nearly 80% of the global crops and acceptance has been growing in many other countries (Jenkins et al., 2021), it is expected that this technology will be a focus of intense development efforts and thus help democratize agricultural biotechnology in the benefit of sustainable food production for the global society.
